# Factorial Reduction of the Main Scales of the Motivated Strategies for Learning Questionnaire (MSLQ) in Mexican Health Sciences University Students

**DOI:** 10.3390/ejihpe15060103

**Published:** 2025-06-05

**Authors:** Aniel Jessica Leticia Brambila-Tapia, Edgar Ulises Velarde-Partida, Laura Arely Carrillo-Delgadillo, Saúl Ramírez-De-los-Santos, Fabiola Macías-Espinoza

**Affiliations:** 1 Departamento de Psicología Básica, Centro Universitario de Ciencias de la Salud (CUCS), Universidad de Guadalajara, Guadalajara 44340, Mexico; ulises.velarde@academicos.udg.mx (E.U.V.-P.); saul.rdelossantos@academicos.udg.mx (S.R.-D.-l.-S.); 2 Licenciatura en Psicología, Centro Universitario de Ciencias de la Salud (CUCS), Universidad de Guadalajara, Guadalajara 44340, Mexico; laura.carrillo4391@alumnos.udg.mx; 3 Departamento de Psicología Aplicada, Centro Universitario de Ciencias de la Salud (CUCS), Universidad de Guadalajara, Guadalajara 44340, Mexico

**Keywords:** MSLQ instrument, Spanish translation, validation, factorial reduction, Mexican population

## Abstract

**Background:** MSLQ is a self-report instrument that measures motivational orientations and learning strategies of college students and is widely used to measure self-regulated learning. MSLQ has not been translated into Spanish and validated in the Spanish-speaking Latin American population. **Objective:** The objective of the study is to adapt, validate, and perform a factorial reduction of 9 out of 15 scales of the MSLQ instrument and correlate the scales with the grade point average (GPA) of a sample of health sciences university students. **Methods:** Nine scales (48 items) of the MSLQ were translated into Spanish and adapted to the Mexican population. Students were invited directly in their classrooms and filled out an electronic questionnaire with personal variables and these nine scales of the MSLQ instrument. We performed exploratory and confirmatory factor analysis (EFA and CFA) and based on the EFA a reduced version of the instrument was proposed. **Results:** A total of 439 students were included. The CFA showed unacceptable fit parameters with the original scale, therefore an item reduction and rearrangement were performed according to the EFA, and this yielded a reduced version with six scales and 25 items which showed acceptable fit parameters. This new reduced version rearranged the items of the effort regulation scale (ERE) into two different scales newly created in this version: time regulation (TRE) and self-regulation (SRE). The scales that disappeared in the reduced version were: intrinsic goal orientation (IGO), meta-cognitive self-regulation (MSR), and elaboration (ELA). **Conclusions:** The reduced version showed acceptable fit parameters that included the creation of two new scales (TRE and SRE). In addition, two scales were reduced (TVA and CTH), three scales were modified (MSE, TSE and ERE), two were unmodified (RHE and ORG), and two scales were eliminated (IGO and ELA).

## 1. Introduction

The motivated strategies for learning questionnaire (MSLQ) is a self-report instrument designed to assess college students’ motivational orientations and their use of different learning strategies for a college course (Duncan et al., 2015; Duncan & McKeachie, 2005). The instrument consists in two sections: a motivation section and a learning strategies section. The motivation section consists in three components: value, expectancy, and affective components, and these three components include six scales: task value (TVA), intrinsic goal orientation (IGO), extrinsic goal orientation (EGO), control beliefs about learning (CLB), self-efficacy for learning and performance (SLP), and test anxiety (TAN). On the other hand, the learning section consists in two components: cognitive and meta-cognitive strategies and resource management strategies; these two components are composed of nine scales: rehearsal (RHE), elaboration (ELA), organization (ORG), critical thinking (CTH), meta-cognitive self-regulation (MSR), time and study environment (TSE), effort regulation (ERE), peer learning (PLE), and help seeking (HSE) (Duncan et al., 2015). Regarding the psychometric properties of the instrument, the originally proposed instrument of 81 items (Duncan et al., 2015; Duncan & McKeachie, 2005) was not validated in terms of statistic indices, and the only fit index used was the GFI which showed a value of 0.78, which is unacceptable (Duncan et al., 2015). Therefore, numerous studies have been performed to validate and/or to reduce and validate the instrument. The last attempt to perform it showed that the 15 specific first-order factors showed the best fit parameters; however, these were still unacceptable for most indices’ estimators used, including the maximum likelihood method (De Araujo et al., 2023). Therefore, the most probable explanation of unacceptable fit parameters in CFA in this and other previous reports (Duncan & McKeachie, 2005; De Araujo et al., 2023; Segura-Robles et al., 2021; Cook et al., 2011; Cook & Skrupky, 2024; Irvine & Williams, 2022) is the inadequate arrangement of items in each scale, which suggest the need to perform new item rearrangements and possible item reduction.

The instrument has been widely used to assess self-regulated learning and has been translated into many languages and validated in different contexts (De Araujo et al., 2023; Segura-Robles et al., 2021; Cook et al., 2011; Cook & Skrupky, 2024; Irvine & Williams, 2022; Nielsen & Hui, 2018). However, the translation of the instrument into the Spanish language has been performed only for two scales (IGO and EGO) (Segura-Robles et al., 2021). In addition, the instrument has been reduced to fewer factors in studies (Cook et al., 2011; Cook & Skrupky, 2024; Irvine & Williams, 2022); nevertheless, the reduction of these factors has not been replicated by different authors and some of these studies were performed only with the components of the motivation section (Cook et al., 2011; Cook & Skrupky, 2024; Dayel et al., 2018; Hamilton & Akhter, 2019). Therefore, the objectives of the present study are: (1) to translate nine of the main scales of the MSLQ instrument into Spanish: TVA, IGO, RHE, ELA, ORG, CTH, MSR, TSE, and ERE. In this translation, the adaptation to culture and target population was considered, and with this objective we expect to perform the translation and cultural adaptation; (2) to apply the instrument to a sample of health sciences university students of Mexico and to perform a confirmatory factor analysis (CFA) with the proposed original version of the instrument, and with this objective we expect to perform the validation of the instrument; (3) to perform an exploratory factor analysis (EFA) with the items included in order to reduce them; (4) to perform a CFA with the reduced scale, and with these two last objectives we expect to perform the factorial reduction of the instrument; and (5) to perform a correlation between the obtained scales and academic achievement, measured by the grade point average (GPA) of the studied students. According to these objectives the hypotheses of the study are: (1) the CFA of the original version of the studied scales shows inadequate fit parameters; (2) the proposed reduced scale based on EFA shows acceptable fit parameters in the CFA; and (3) all the reduced scales show positive and significant correlations with GPA.

## 2. Materials and Methods

### 2.1. Ethical Considerations

The study was conducted according to the guidelines of the Declaration of Helsinki and was approved by the ethical committee of the Health Sciences University Center, with the registration number CI-06022 (approval date 30 September 2022). All personal and health-related data of participants were handled with strict confidentiality, being used only for research purposes. This study is part of another project about which a publication has already been published (Brambila-Tapia et al., 2024). Clinical trial number: not applicable. All the participants signed an informed consent form.

### 2.2. Subjects

The inclusion criteria of the study were: (a) active students of the Health Sciences University Center of the University of Guadalajara (all of them of mestizo ethnical origin), (b) students older than 18 years old, (c) those who accepted to participate in the study and signed an informed consent form. The exclusion criterion was: (a) those students who did not complete the measurement of all the instruments and tests. These inclusion criteria are the same as those used in a previous publication derived from the same project (Brambila-Tapia et al., 2024). However, that project had many objectives and one of them was the validation and factorial reduction of the MSLQ scale, therefore, this study complies with that specific objective, and no analyses overlapping with the previous report of the research team are present (Brambila-Tapia et al., 2024).

A total of 439 students were included, of whom 297 (67.7%), were women, the mean ± SD of age was: 20.34 ± 2.61 years old, with a range of 18–54 years. Most students had a preparatory level (93.1%) and a medium socioeconomic level (78.7%). The participants were studying one of 6 different bachelor’s programs in health sciences, including: nursing, physical culture and sport, medicine, psychology, dental prothesis, and higher university technician in emergencies, work safety, and rescues.

### 2.3. Study Design and Procedures

This is a cross-sectional and instrumental design (Ato et al., 2013) as the study has the purpose to analyze the psychometric properties of an instrument and the measures were performed in only one moment.

Health sciences university students were invited to participate directly in their classrooms. Those who accepted were invited to a computational room of the Health Sciences University Center of the University of Guadalajara, where they signed an informed consent form and filled out an electronic questionnaire with sociodemographic variables and the studied scales of the MSLQ scale. The measurement of these variables lasted around 1 h and no compensation was given to the participants. The GPA of each student was obtained from institution reports. The study was performed from October to December of 2022.

### 2.4. Instrument

The MSLQ was developed and validated in the English language by Duncan & McKeachie (Duncan & McKeachie, 2005), the answer options are Likert type (1 = not at all, 2 = to a small degree, 3 = to some degree, 4 = to a large degree, 5 = perfectly), and the instrument consists in two large sections: motivation and learning strategies, including 15 different scales. In this study we included 9 of the total 15 scales, 2 motivation scales: TVA and IGO, and 7 learning strategies scales: RHE, ELA, ORG, CTH, MSR, TSE, and ERE. In the case of the MSR scale we only included 7 out of the 12 items, which were chosen by the research team based in their clarity and potential importance for academic achievement. The items included in English and Spanish are described in Supplementary File S1. The included scales were chosen based on their potential utility to predict academic achievement. In this sense, 4 scales of the motivation section (EGO, CLB, SLP, and TA) that were not included were considered tangential and not directly related to intrinsic motivation or to a personal interest in the study subject, as were the included IGO and TVA scales. Likewise, the excluded scales of the learning strategies section (HSE and PLE) were considered external studying strategies as they involve other people for help seeking and peer learning.

### 2.5. Instrument Translation

The selected items were translated and retro-translated to Spanish and English by different bilingual experts in each process (translation and retro-translation) in order to detect inconsistencies, then the final version was reviewed by the research team, which proposed some modifications. These were incorporated into the final translation which was performed by the bilingual experts and approved by the research team. For the Spanish translation, the local culture, including the student characteristics of the University of Guadalajara, was considered. In this sense, the translated questions were shown to a group of students in order to determine their comprehensibility. The items in English and Spanish are mentioned in Supplementary File S1.

### 2.6. Variables

The sociodemographic variables measured were: age, sex, schooling, socioeconomic level, and the bachelor’s program that they were studying.

The studying strategies of the MSLQ included 2 motivation scales: TVA and IGO, and 7 learning strategies scales: RHE, ELA, ORG, CTH, MSR, TSE, and ERE.

### 2.7. Statistical Analysis

For descriptive values of quantitative variables including sociodemographic and instrument variables we used means and standard deviations and median and ranges for parametric and non-parametric distributions, respectively. Qualitative variables were described with frequencies and percentages. To perform EFA, CFA, and the McDonald’s omega test we used JASP software version 0.16.4 (Intel) (JASP Team, 2022). In the EFA we obtained the factor load using orthogonal varimax rotation analysis assuming that factors are correlated and the estimator method was the minimum residual. For CFA we obtained the comparative fix index (CFI) with the maximum likelihood (ML) estimator method, considering it the more reliable estimator method for CFA. We also obtained the Tucker–Lewis index (TLI), the standardized root mean square residual (SRMS), and the root mean square error of approximation (RSMA). Values of CFI and TLI > 0.90 and values of SRMS and RSMA < 0.08 are considered good fit indices for a model (Baumgartner & Homburg, 1996). We also obtained the Cronbach’s alpha and performed McDonald’s omega tests for each scale of the instrument, with a value > 0.70 considered acceptable (Montazeri et al., 2009). The correlations between the studying strategies with the GPA were performed with Pearson and Spearman correlation tests, depending on whether the distribution of the data was parametric or non-parametric, respectively. Descriptive statistics, correlations, and Cronbach’s alpha tests were performed with SPSS v.25 software.

## 3. Results

### 3.1. CFA and EFA

The CFA for the original model with the nine scales included as first-order factors and assuming the factors correlated yielded a model with an unacceptable fit, with a CFI: 0.683, TLI: 0.665, and RMSEA: 0.078.

The EFA, with the studied items, yielded six different factors, of which four included the complete items of the same scale: TVA, CTH, ORG and RHE, and the fifth and sixth ones represented a combination of two different scales: ERE and TSE for factor 5 (this new scale was named time regulation (TRE)), and MSR and ERE for factor 6 (this new scale was named self-regulation (SRE)).

We observed that most of the items of the ELA scale loaded in the same factor of CTH items but with lower loading values than CTH items. The IGO scale did not load in the same factor alone or in combination with other factors, therefore these scales were not considered in the newly proposed reduced version. The model figure of the CFA is shown in Supplementary File S2.

### 3.2. CFA for the Reduced Scale

To create the reduced version of the MSLQ instrument, we selected the first four items of each scale, which had the highest loads values for TVA and CTH. For the combined scales (TRE and SRE) which were the newly proposed scales, we selected at least four items based in the highest loads in the EFA and in their item similitude. In this sense for TRE, four items were included in it, two from the ERE scale and two from the TSE scale, and for the SRE scale, five items were included, two from the ERE scale and three from the MSR scale, and with this item organization we then performed the CFA for the reduced version.

The CFA for the reduced scale yielded acceptable fit parameters, and the fit parameters and item load of the reduced scale obtained in the CFA are presented in Table 1 and Table 2. To select the items in the reduced version we included the items with the highest factorial load in the EFA and their correspondence with the item number of original scales is mentioned in Table 2.

All the Cronbach’s alpha and McDonald’s omega tests for each factor yielded desirable values and are included in Table 2.

With the reduced version of the instrument (Table 2), we diminished the number of items from the 48 items included in the study, which represented nine scales of the original MSLQ instrument, to 25 items in six scales. It is noticeable that two new scales were created based in the EFA and the items similitude, therefore, two items of the ERE scale were moved to the newly created TRE scale, which seems to be more specific to measure the regulation of time of study (TRE), and the other two items of the ERE scale were moved to the newly created SRE scale which measures the student’s abilities in adapting their cognitive abilities in understanding the course material and their effort regulation in the face of uninteresting tasks (SRE). Two scales of the new version remained the same (ORG and RHE), and this was performed because, according to the EFA, the items of these scales loaded in the same factor, and these scales had only four items. The Spanish translation of the proposed items in this reduced version is mentioned in Table 3.

### 3.3. Correlations Between MSLQ Scales and GPA

In Table 4 we present the correlations between the six scales of the reduced version and the GPA of the bachelor’s students and their comparison with the nine original scales studied in this report, and we can observe that correlation values with the new reduced and modified scales were very similar to the original scales.

In Table 5 we show the covariances of the CFA between the factors of the reduced scale, where we can observe that all of them are statistically significant.

## 4. Discussion

We corroborated all three hypotheses of the study. The CFA performed with the original version of the instrument did not show acceptable fit parameters, however, the reduced version based on EFA did show acceptable fit parameters in the CFA. Finally, according to the third hypothesis all the reduced scales of the new version of the MSLQ instrument showed significant positive correlations with GPA.

In this study, we report the Spanish adaptation, validation, and factorial reduction of most scales of the MSLQ instrument and an item reduction of around 50% of the items investigated. In addition, the nine scales included in this report were reduced to six, where two scales were created (TRE and SRE), two were reduced (TVA and CTH), two remained the same (ORG and RHE), three were modified (MSR, TRE, and ERE), and two were eliminated (ELA and IGO).

In relation to the CFA performed on the original version we observed that the model fit was unacceptable, and this result coincides with previous reports where unacceptable values of the CFA were observed with this original version (De Araujo et al., 2023; Segura-Robles et al., 2021; Cook et al., 2011; Cook & Skrupky, 2024; Irvine & Williams, 2022; Alkjarusi et al., 2012). Even the CFA performed on the original version by the scale developers showed unacceptable fit parameters for both motivation and strategies scales (Duncan et al., 2015). These results indicate that some items do not represent the specific scale where they were originally placed or that some items do not represent any scale of the instrument. For these cases, EFA is very useful, however, few studies have been performed with this purpose (Irvine & Williams, 2022) and none of them was performed in Spanish and specifically in a Latin American population.

In this report, we detected that, by selecting the items with the highest loading values of CTH, ORG, TVA, and RHE scales, these scales could be reliably measured with 16 items, 4 for each one. In the case of CTH we also observed that, according to EFA, CTH also measured the abilities of the ELA scale. This is because most ELA items loaded in the CTH factor but with lower scores, therefore, it is assumed that the CTH factor also measures ELA abilities. This possibility seems plausible because the theoretical model mentions that ELA abilities are related to creating connections between the information to be learned, which is also related with CTH abilities, and this includes the application of previous knowledge to new situations, therefore the ELA scale was not included in the present model by assuming that it can be reliably measured with CTH scale and because CTH items showed higher loading values in EFA than ELA items. Another scale that is not included in the reduced version of the instrument is ERE. In this case, the EFA grouped its items with items of TSE and MSR scales and, after analyzing the items and by considering that half of the ERE scale (two items) grouped with another two items of the TSE scale and the other two items of the ERE scale grouped with three items of the MSR scale, we decided to modify these scales, renaming them TRE and SRE. These scales better represent the time dedicated to study (items TRE2 and TRE3) and the effort put in at this time (items TRE1 and TRE4), in the case of TRE scale, and the monitoring and regulation in learning the information of the courses, in the case of SRE. The item of monitoring included in the revised scale is SRE2, while SRE3 is a regulation item and SRE4 a planning item. Finally, the items SRE1 and SRE5 correspond to the ERE items of the original version that in our opinion also belong to regulation activities, as the effort performed by the student in order to adapt to boring or difficult activities, therefore, the SRE scale refers to activities related to self-regulation in studying activities, including planning, monitoring, and effort. It is important to mention that the rest of the items included in the original scale of TSE, many of them dedicated to the study environment, also loaded in the same factor of the newly created TRE scale but with lower loading values, while the rest of the items did not load in any factor.

The IGO scale neither loaded in the same factor nor was included in the revised version. The scales excluded or modified in this reduced version (IGO, MSR, and ELA) did not show an acceptable reliability, measured with Cronbach’s alpha tests and McDonald omega tests, in a previous report (De Araujo et al., 2023). However, in this report, the six proposed factors (scales) were reliable in terms of Cronbach’s alpha and the CFA model fit parameters. It was also observed that the ERE scale had previously shown a Cronbach’s alpha below 0.70 (Duncan et al., 2015), as was observed in the present study (Supplementary File S1), but with the modifications performed in this version all the scales had a Cronbach’s alpha above 0.70. We also observed that the correlations with GPA were similar to those observed with the original scales and were also similar to the correlations previously reported between MSLQ scales and GPA (Credé & Phillips, 2011). However, with the modifications performed in the reduced scales we observed that SRE was the scale with the highest correlation with GPA, followed by TRE and TVA; this is different to the correlations with the original scales, where ERE was the scale with the highest correlation with GPA, followed by TSE. These changes are explained by the fact that ERE items were rearranged with SRE and TRE scales. Although the correlations between the instrument scales and GPA are significant, they are low, with correlation coefficients ranging from 0.09–0.27. These correlations coincide with previous reports (Credé & Phillips, 2011), which indicates that these results are reliable, however, the low correlation coefficients also indicate that GPA could be modestly influenced by studying strategies. This point is sustained with the results previously observed by the research team, where GPA was associated by many variables, one of them being the studying strategies, which played a modest although significant role, as observed in the multivariate regression analyses for GPA (Brambila-Tapia et al., 2024).

We also observed that all the reduced scales showed significative covariances among them, which coincides with the fact that these scales are part of the same instrument and contribute to increased GPA and grades. In addition, we observed that the inter-factor covariances were similar with respect to the reported inter-correlations in the original scale (Duncan et al., 2015; Chow & Chapman, 2017).

The Spanish translated and reduced version of the MSLQ instrument implies a simplified method to measure studying strategies in Spanish-speaking populations, which can also be verified in English and other languages. The use of this scale would increase the knowledge on the relationship between studying strategies and academic achievement and other academic factors in different populations. It is remarkable that the MSLQ instrument has been studied in different languages and contexts with a special emphasis in medical or health sciences students (Zilundu et al., 2021; Fatima et al., 2025); however, few reduction attempts have been performed and none in a Spanish-speaking population, a gap that this study attempts to fill.

The main limitation of the study is the inclusion of only 9 out of the 15 scales of the MSLQ instrument, which diminishes the ability to rearrange the items of the other unmeasured scales. In addition, for the MSR scale, of the 12 original items we only selected 7 based on their clarity and potential importance for academic achievement. With respect to the unmeasured scales (EGO, CLB, TA, SLP, HSE, and PLE), we observed that some of them were negatively correlated with GPA (HSE, PLE, and TA) or presented low positive correlations with it (EGO and CLB), and only the SLP scale showed similar correlations with GPA to those observed in this study (rho = 0.21) (Credé & Phillips, 2011). However, this scale measures a student’s self-confidence in their study abilities, which could not be considered a studying strategy by itself. On the other hand, the main strengths of the study are the Spanish adaptation of MSLQ into a Latin American context and its factorial reduction and item rearrangement that improves CFA fit parameters, reduces the number of items, and increases the specificity of them to each scale.

## 5. Conclusions

In conclusion, we present an adaptation to Spanish of the main scales of the MSLQ instrument with a factorial reduction and item rearrangement that reduces the time of application and increases the precision of the instrument in order to measure specific motivational and studying strategies that show positive correlations with GPA in Mexican bachelor’s students. Further studies that measure this new version in different languages and contexts are needed to corroborate these results.

## Figures and Tables

**Table 1:** CFA fit parameters and factor load of the items in the reduced MSLQ scale.

CFI	TLI	RMSEA	Xi^2^ (df)	*p* Value
0.926	0.915	0.049	3989.112 (300)	<0.01

df: degrees of freedom.

**Table 2:** Factor load of the CFA of each item included in the reduced version of MSLQ.

Item	Factor 1	Factor 2	Factor 3	Factor 4	Factor 5	Factor 6
CTH_1 (item 51)	0.976					
CTH_2 (item 47)	0.700					
CTH_3 (item 66)	0.698					
CTH_4 (item 71)	0.903					
ORG_1 (item 49)		0.924				
ORG_2 (item 63)		0.807				
ORG_3 (item 32)		0.871				
ORG_4 (item 42)		0.928				
TVA_1 (item 17)			0.573			
TVA_2 (item 23)			0.629			
TVA_3 (item 10)			0.636			
TVA_4 (item 27)			0.579			
TRE_1 (item 37)				1.008		
TRE_2 (Item 77)				0.746		
TRE_3 (item 52)				0.718		
TRE_4 (item 60)				0.699		
RHE_1 (item 39)					0.772	
RHE_2 (item 46)					0.757	
RHE_3 (item 59)					0.761	
RHE_4 (item 72)					0.748	
SRE_1 (item 74)						0.626
SRE_2 (item 41)						0.530
SRE_3 (item 44)						0.707
SRE_4 (item 54)						0.583
SRE_5 (item 48)						0.607
Cronbach’s alpha/McDonald’s omega	0.816/0.818	0.803/0.804	0.849/0.848	0.708/0.714	0.756/0.756	0.704/0.706

CTH: Critical thinking, ORG: Organization, TVA: Task value, TRE: Time regulation, RHE: rehearsal, SRE: Self-regulation. Item numbers correspond to the original scale (Duncan et al., 2015).

**Table 3:** Items included (English and Spanish) in each scale of the reduced instrument.

Item	English/Spanish
CTH1	I treat the course material as a starting point and try to develop my own ideas about it/“Tomo el material de clase como punto de partida y trato de desarrollar mis propias ideas sobre el tema”
CTH2	When a theory, interpretation, or conclusion is presented in class or in the readings, I try to decide if there is good supporting evidence/“Cuando se presenta una teoría, interpretación o conclusión en clase, trato de decidir si hay buena evidencia de apoyo”
CTH3	I try to play around with ideas of my own related to what I am learning in this course/“Trato de jugar con mis propias ideas relacionadas con lo que estoy aprendiendo en clase”
CTH4	Whenever I read or hear an assertion or conclusion in this class, I think about possible alternatives/“Cada vez que escucho una afirmación o conclusión en clase pienso en posibles alternativas”
ORG1	I make simple charts, diagrams, or tables to help me organize course material/“Realizo gráficos, diagramas o tablas simples para ayudarme a organizar la información”
ORG2	When I study for this course, I go over my class notes and make an outline of important concepts/“Cuando estudio para mis clases reviso mis notas y hago un resumen de los conceptos importantes”
ORG3	When I study the readings for this course, I outline the material to help me organize my thoughts/“Cuando estudio para mis clases, bosquejo la información para ayudarme a organizar mis pensamientos”
ORG4	When I study for this course, I go through the readings and my class notes and try to find the most important ideas/“Cuando estudio para mis clases, reviso las lecturas y notas de clase y trato de encontrar las ideas más importantes”
TVA1	I am very interested in the content area of this course/“Estoy muy interesado en el contenido de mis clases”
TVA2	I think the course material in this class is useful for me to learn/“Creo que la información de mis clases es útil para que la aprenda”
TVA3	It is important for me to learn the course material in this class/“Es importante para mí aprender la información de mis clases”
TVA4	Understanding the subject matter of this course is very important to me/“Es muy importante para mí comprender la información de mis clases”
TRE1	I often feel so lazy or bored when I study for this class that I quit before I finish what I planned to do/“A menudo me siento con tanta flojera o aburrimiento cuando estudio para mis clases que dejo de hacerlo antes de lo planeado”
TRE2	I often find that I don’t spend very much time on this course because of other activities/“A menudo encuentro que no le dedico mucho tiempo a mis clases debido a otras actividades” (Reversed)
TRE3	I find it hard to stick to a study schedule/“Me resulta difícil cumplir con un horario de estudio” (Reversed)
TRE4	When course work is difficult, I give up or only study the easy parts/“Cuando el trabajo de las clases es difícil, me doy por vencido o solo estudio las partes fáciles”
RHE1	When I study for this class, I practice saying the material to myself over and over/“Cuando estudio para mis clases practico diciendo la información varias veces”
RHE2	When studying for this class, I read my class notes and the course readings over and over again/“Cuando estudio para mis clases leo las notas y lecturas varias veces”
RHE3	I memorize key words to remind me of important concepts in this class/“Memorizo palabras clave para recordar conceptos importantes de mis clases”
RHE4	I make lists of important terms for this course and memorize the lists/“Hago listas de términos importantes para mis clases y los memorizo”
SRE1	Even when course materials are dull and uninteresting, I manage to keep working until I finish/“Incluso cuando los temas del curso son aburridos o poco interesantes, sigo trabajando hasta que termino”
SRE2	When I become confused about something I’m reading for this class, I go back and try to figure it out/“Cuando me confundo con algo que estoy leyendo para las clases, regreso al punto y trato de resolverlo”
SRE3	If course materials are difficult to understand, I change the way I read the material/“Si los temas del cuso son difíciles de entender cambio la forma en que leo el material”
SRE4	Before I study new course material thoroughly, I often skim it to see how it is organized/“Antes de estudiar a fondo nueva información, la hojeo para ver cómo está organizada”
SRE5	I work hard to do well in this class even if I don’t like what we are doing/“Trabajo duro para que me vaya bien en las clases, incluso si no me gusta lo que estamos haciendo”

**Table 4:** Correlations between the reduced scales and grade point average (GPA).

	Scales	GPA
Reduced scales	Critical thinking (CTH)	0.11 *
	Organization (ORG)	0.21 **
	Task value (TVA)	0.21 **
	Time regulation (TRE)	0.23 **
	Rehearsal (RHE)	0.14 **
	Self-regulation (SRE)	0.26 **
Original scales	Critical thinking (CTH)	0.12 *
	Organization (ORG)	0.21 **
	Task value (TVA)	0.24 **
	Time study and environment (TSE)	0.27 **
	Effort regulation (ERE)	0.28 **
	Elaboration (ELA)	0.23 **
	Intrinsic goal orientation (IGO)	0.09
	Meta-cognitive self-regulation (MSR)	0.18 **
	Rehearsal (RHE)	0.14 **

*p* values obtained with Spearman correlation tests. * *p* < 0.05, ** *p* < 0.01.

**Table 5:** Covariances between the reduced scales.

Scales	TVA	ORG	TRE	CTH	SRE	RHE
TVA	-	0.22 **	0.21 **	0.18 **	0.39 **	0.47 **
ORG		-	0.45 **	0.43 **	0.64 **	0.58 **
TRE			-	0.23 **	0.49 **	0.31 **
CTH				-	0.53 **	0.22 **
SRE					-	0.42 **
RHE						-

CTH: Critical thinking, ORG: Organization, TVA: Task value, TRE: Time regulation, RHE: rehearsal, SRE: Self-regulation. *p* values obtained with the covariances of CFA. ** *p* < 0.01.

## Data Availability

The datasets used and/or analyzed during the current study are available from the corresponding author on reasonable request.

## Supplementary Materials

The following supporting information can be downloaded at: https://www.mdpi.com/article/10.3390/ejihpe15060103/s1, Figure S1: Model Figure of the CFA of the reduced version, Table S1: MSLQ items included in Spanish and English.
